# Effect of multi-disciplinary team care program on quality of life, anxiety, and depression in hepatocellular carcinoma patients after surgery: A randomized, controlled study

**DOI:** 10.3389/fsurg.2022.1045003

**Published:** 2023-01-06

**Authors:** Li Yang, Chenli Yan, Jingjing Wang

**Affiliations:** Department of Hepatobiliary and Pancreatic Surgery, The Central Hospital of Wuhan, Tongji Medical College, Huazhong University of Science and Technology, Wuhan, China

**Keywords:** multi-disciplinary team care program, quality of life, anxiety, depression, hepatocellular carcinoma

## Abstract

**Objective:**

Multi-disciplinary team (MDT) collaboration enables hepatocellular carcinoma (HCC) patients to achieve better survival through precise diagnosis and individualized treatment. This study aimed to further investigate the effect of MDT care program (MDT-CP) on quality of life (QoL), anxiety and depression in HCC patients after surgery.

**Methods:**

Totally, 150 postoperative HCC patients were enrolled and randomized in a 1:1 ratio into the MDT-CP group (*N* = 76) to receive MDT care for 6 months and the normal care program (N-CP) group (*N* = 74) to receive routine care for 6 months.

**Results:**

Quality of Life Questionnaire-Core 30 (QLQ-C30) global health status score at 1 month (M1), M3 and M6, QLQ-C30 functions score at M3 and M6 elevated while QLQ-C30 symptom score at M1 and M3 decreased in MDT-CP group compared with N-CP group (all *P* < 0.05). In addition, Hospital Anxiety and Depression Scale (HADS)-Anxiety score at M3 and M6, anxiety occurrence rate at M6, anxiety degree at M6, were all reduced in MDT-CP group compared with N-CP group (all *P* < 0.05). HADS-Depression score at M6, and depression occurrence rate at M3, were both lessened in MDT-CP group compared to N-CP group (both *P* < 0.05), while there was no distinction of depression degree at any time points between groups.

**Conclusion:**

MDT-CP improves QoL, relieves anxiety and depression to a certain extent in HCC patients after surgery.

## Introduction

Hepatocellular carcinoma (HCC) is the most common form of liver cancer and accounts for 8.3% of the world's cancer deaths (1–3). Studies have identified the major risk factors for HCC, including Hepatitis B virus (HBV), hepatitis C virus, alcoholic and nonalcoholic fatty liver disease (4). In addition, co-infection of hepatitis D virus (HDV) and HBV significantly increases the risk of HCC recurrence in patients undergoing living donor liver transplantation (5). Together with the progresses in early detection and treatment of HCC, the morbidity and mortality of HCC have been reduced to some extent (6). However, HCC still imposes a huge financial and life burden on patients (7). These burdens often reduce quality of life (QoL) and cause anxiety and depression in HCC patients.

The nursing management of QoL, anxiety and depression in HCC patients is highly concerned in recent years (8, 9). One recent study illustrates that the model of 5A nursing intervention is a new nursing model that offers nursing intervention through inquiry, suggestion, evaluation, help, and follow-up with patients, which could elevate QoL in HCC patients after surgery (10). Meanwhile, a comprehensive education and care program improves QoL, anxiety and depression, and prolongs survival in patients with HCC undergoing surgical resection (11). In addition, our previous study also illustrates reminiscence therapy-based care ameliorates QoL, anxiety and depression in elderly patients with HCC (12). Considering that poor QoL, anxiety and depression are still prevalent and positively associated with HCC mortality (13–15), it is necessary to explore novel intervention to manage the QoL, anxiety, and depression in HCC patients.

Multi-disciplinary team (MDT) is the collaboration of multiple specialists from different disciplines to achieve coordinated management (16), which aims to delay disease progression, prolong survival, and maintain an acceptable QoL in patients (17). A previous study shows MDT collaborative nursing model can elevate QoL, alleviate anxiety, depression and other negative emotions in the postoperative advanced pancreatic cancer patients (18). Moreover, other studies illustrate that MDT also improve QoL, anxiety, depression in patients with cervical cancer and gastric cancer (19, 20). However, the impact of MDT management on QoL and mental health in HCC patients is still unclear.

Thus, we designed an MDT care program (MDT-CP) in this randomized, controlled study, aiming to compare the effect of MDT-CP versus normal care program (N-CP) on QoL and mental health in HCC patients after surgery.

## Methods

### Patients

A total of 150 primary HCC patients who underwent tumor resection from February 2019 to March 2021 were consecutively enrolled in this randomized, controlled study. The inclusion criteria contained: (a) pathologically diagnosed as HCC; (b) older than 18 years; (c) underwent tumor resection; (d) were voluntary for participation and willing for complying with the study protocol. The exclusion criteria contained: (a) had other malignancies; (b) complicated with a severe mental disorder or cognitive impairment that unable to comply with the study assessment; (c) had psychological intervention history. The study was permitted by Ethics Committee of The Central Hospital of Wuhan, Tongji Medical College, Huazhong University of Science and Technology. Written informed consent was gained from each patient.

### Random assignment

Included patients were randomly assigned according to a 1:1 ratio using the block randomization method with a block size of 4 (21). Briefly, scratch cards were made to record patients’ randomization information and were distributed to patients when they were deemed eligible for inclusion. Based on the first 37 blocks, patients were assigned to two groups with 74 patients in each group; while based on the last block (38th block), 2 patients were both assigned to the MDT-CP group. Therefore, the included patients were assigned to two groups: 76 in the MDT-CP group and 74 in the N-CP group.

### Intervention

For the N-CP group, routine care was provided, and routine verbal and written discharge instructions were given, including dietary and medication instructions and precautions related to HCC. For the first 6 months after discharge, patients were regularly admitted to the hospital for follow-up once a month. During the follow-up period, the patient's condition was monitored and health guidance was provided.

For the MDT-CP group, patients received MDT-CP on the basis of normal care, and the specific methods are as follows: (a) a multi-disciplinary team was formed, consisting of 2 attendings from the department of hepatobiliary and pancreatic surgery, 2 nutritionists from nutrition department, 2 rehabilitation physicians from rehabilitation department, 2 psychiatrists from psychiatry department, and 4 nursing staff from nursing department; (b) an MDT-CP was developed: a thorough assessment of the patient's physical, psychological, environmental, and health behaviors was conducted, then a rational plan of care was discussed with the patient's family to clarify the patient's needs; (c) the program was implemented: patients were followed up weekly by telephone and monthly by home visits or outpatient visits, during the follow-up, the following nursing measures were implemented according to the patient's actual condition: (1) the department of hepatobiliary and pancreatic surgery was mainly responsible for observing patients’ physiological indications, changes in their condition, recovery of liver function, and postoperative functional recovery, in order to improve patient's condition and promote disease recovery; (2) the nutrition department was responsible for developing a nutritional plan and encouraging patients to have a rational diet; (3) the rehabilitation department was responsible for developing a training plan based on the patient's physical condition and tolerance level; (4) the psychiatry department was responsible for psychological intervention according to the patient's condition, encouraging the patient to communicate emotionally and relieving the patient's bad mood; (5) the nursing department was responsible for training relevant staff and supporting the whole program (22, 23).

### Follow-up and assessment

Following the patient's discharge from the hospital, outpatient or telephone follow-up was performed for 6 months. Quality of Life Questionnaire-Core 30 (QLQ-C30) and Hospital Anxiety and Depression Scale (HADS) scores were assessed on discharge (M0), at 1 month after discharge (M1), at 3 months after discharge (M3), and at 6 months after discharge (M6). The QLQ-C30 score was used to assess patients’ quality of life (24). The HADS scale was used to assess anxiety and depression, with no being 0–7 scores, mild being 8–10 scores, moderate being 11–14 scores, and severe being 15–21 scores (25).

### Statistics

The sample size was determined by assuming that the mean QLQ-C30 global health status score for the MDT-CP group was 85 with a standard deviation (SD) of less than 20 and the mean QLQ-C30 global health status score for the N-CP group was 75 with an SD of less than 20 at M6 (26). The minimum sample size for each group was 63 at a significance (*α*) level of 0.05 and a power of 85%, which was then adjusted for a 15% dropout rate and increased to 73. SPSS v.26.0 (IBM Corp., America) was used for analysis. GraphPad Prism v.9.0 (GraphPad Software Inc., America) was used for graphing. Kolmogorov-Smirnov test was utilized for normality determination. The normal distributed continuous variables (age, QLQ-C30 score, and HADS score) were displayed as mean ± SD and analyzed using Student's *t*-test. The skewed distributed continuous variables (liver function indexes) were presented as median and inter-quartile range (IQR) and analyzed using Mann-Whitney *U* test. The binary categorized variables and unordered categorical variables (gender, smoke, marriage status, employment status before surgery, location, chronic comorbidities, ECOG PS score, Child-Pugh stage, tumor nodule number, largest tumor size, anxiety occurrence, and depression occurrence) were expressed as count (percentage) and analyzed using Chi-Square test. The ordinal categorical variables (level of education, BCLC stage, CNLC stage, anxiety degree, and depression degree) were expressed as count (percentage) and analyzed using Mann-Whitney *U* test. *P* < 0.05 was considered significant.

## Results

### Study flow

**Figure 1:**
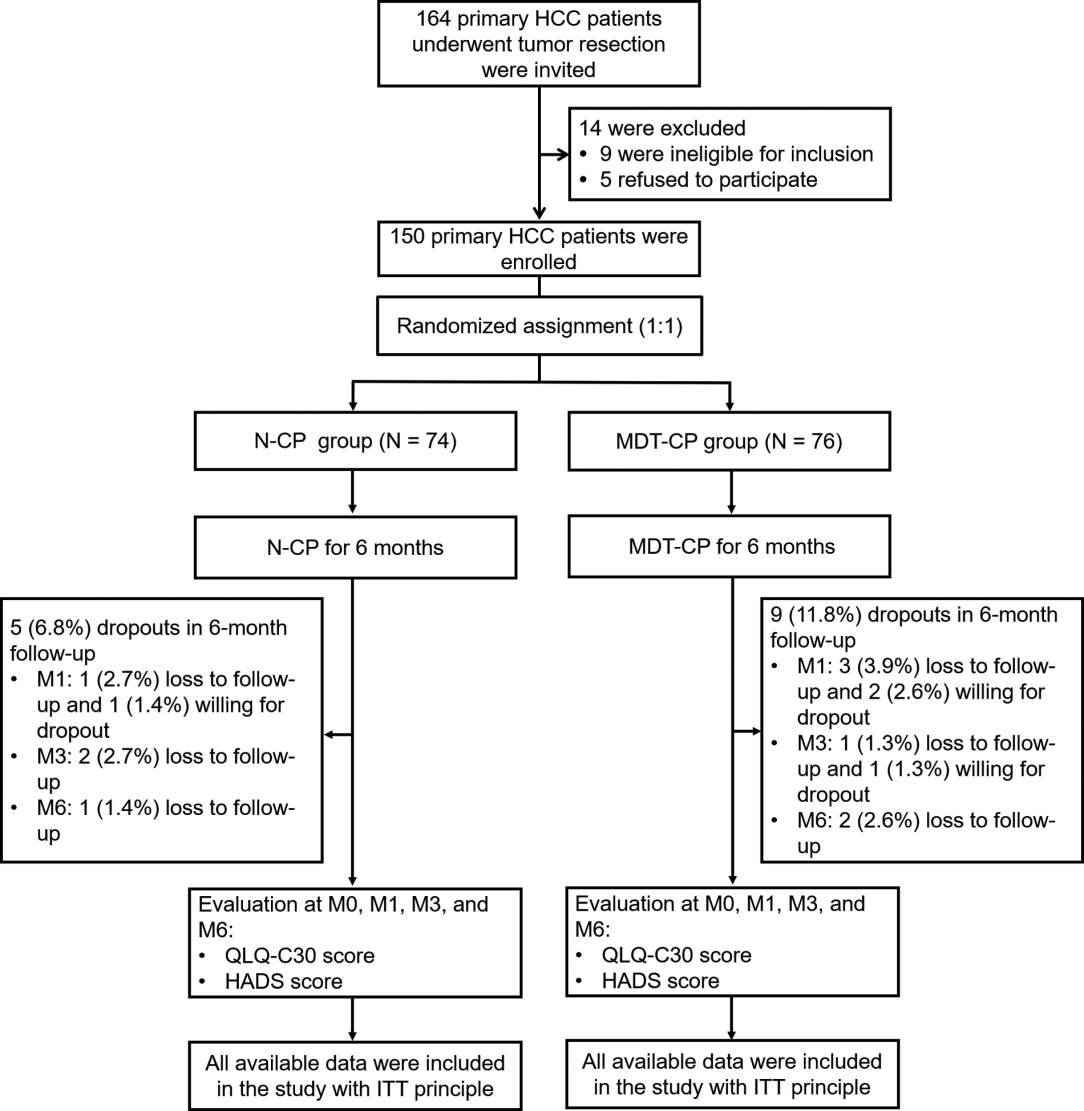
Study flow chart.

A total of 164 primary HCC patients who underwent tumor resection were invited, while 14 of them were excluded from this study, including 9 patients who either met the exclusion criteria or did not meet the inclusion criteria, and 5 patients who refused to participate. The remaining 150 patients were then randomized as 1:1 ratio into N-CP group (*N* = 74) and MDT-CP group (*N* = 76) to receive N-CP and MDT-CP interventions for 6 months, respectively. During follow-up, 5 (6.8%) patients in N-CP group withdrew, including 1 (1.4%) patient who lost to follow-up and 1 (1.4%) patient who was willing for dropout at M1, 2 (2.7%) patients who lost to follow-up at M3, and 1 (1.4%) patient who lost to follow-up at M6. In MDT-CP group, 9 (11.8%) patients withdrew, which included 3 (3.9%) patients who lost to follow-up and 2 (2.6%) patients who was willing for dropout at M1, 1 (1.3%) patient who lost to follow-up and 1 (1.3%) patient who was willing for dropout at M3, and 2 (2.6%) patients who lost to follow-up at M6. In both groups, QLQ-C30 and HADS scores were evaluated at M0, M1, M3, and M6, respectively. Finally, all available data were included with ITT principle (Figure 1).

### Comparison of baseline characteristics between groups

The mean age was 57.4 ± 9.8 and 56.8 ± 9.3 years respectively in MDT-CP and N-CP groups. Meanwhile, there were 8 (10.8%) females in MDT-CP group and 12 (15.8%) females in N-CP group, accordingly. Further comparison analysis showed that no discrepancy was observed in clinical characteristics, including demographic characteristics, chronic comorbidities, and disease features between MDT-CP and N-CP group (all *P* > 0.05) (Table 1). Moreover, it was found that there was no difference in the level of liver function indexes and tumor markers between MDT-CP group and N-CP group (all *P* > 0.05) (Table 2).

**Table 1:** Clinical characteristics.

Items	N-CP group (*N* = 74)	MDT-CP group (*N* = 76)	*P* value
**Demographics**			
Age (years), mean ± SD	56.8 ± 9.3	57.4 ± 9.8	0.696
Gender, No. (%)			0.370
Female	8 (10.8)	12 (15.8)	
Male	66 (89.2)	64 (84.2)	
Smoke, No. (%)			0.944
Never	32 (43.2)	33 (43.4)	
Former	18 (24.3)	20 (26.3)	
Current	24 (32.4)	23 (30.3)	
Marriage status, No. (%)			0.771
Married	57 (77.0)	57 (75.0)	
Single/Divorced/Widowed	17 (23.0)	19 (25.0)	
Employment status before surgery, No. (%)			0.750
Employed	36 (48.6)	35 (46.1)	
Unemployed	38 (51.4)	41 (53.9)	
Level of education, No. (%)			0.712
Primary school or less	8 (10.8)	5 (6.6)	
High school	36 (48.6)	43 (56.6)	
Undergraduate	24 (32.4)	22 (28.9)	
Graduate or above	6 (8.1)	6 (7.9)	
Location, No. (%)			0.658
Rural	8 (10.8)	10 (13.2)	
Urban	66 (89.2)	66 (86.8)	
**Chronic comorbidities**			
History of HB, No. (%)	61 (82.4)	61 (80.3)	0.733
History of liver cirrhosis, No. (%)	53 (71.6)	57 (75.0)	0.640
History of hypertension, No. (%)	26 (35.1)	31 (40.8)	0.476
History of hyperlipidemia, No. (%)	12 (16.2)	19 (25.0)	0.184
History of diabetes, No. (%)	7 (9.5)	12 (15.8)	0.244
**Disease features**			
ECOG PS score, No. (%)			0.927
Score 0	56 (75.7)	58 (76.3)	
Score 1	18 (24.3)	18 (23.7)	
Child-pugh stage, No. (%)			0.938
Stage A	60 (81.1)	62 (81.6)	
Stage B	14 (18.9)	14 (18.4)	
Tumor nodule number, No. (%)			0.255
Unifocal	35 (47.3)	43 (56.6)	
Multifocal	39 (52.7)	33 (43.4)	
Largest tumor size, No. (%)			0.191
<5.0 cm	41 (55.4)	34 (44.7)	
≥5.0 cm	33 (44.6)	42 (55.3)	
BCLC stage, No. (%)			0.754
Stage 0	0 (0.0)	1 (1.3)	
Stage A	34 (45.9)	37 (48.7)	
Stage B	22 (29.7)	20 (26.3)	
Stage C	18 (24.3)	18 (23.7)	
CNLC stage, No. (%)			0.422
Stage Ia	13 (17.6)	9 (11.8)	
Stage Ib	27 (36.5)	36 (47.4)	
Stage IIa	22 (29.7)	23 (30.3)	
Stage IIb	12 (16.2)	8 (10.5)	

N-CP, normal care program; MDT-CP, multi-disciplinary team care program; SD, standard deviation; HB, hepatitis B; ECOG PS, eastern cooperative oncology group performance status; BCLC, barcelona clinic liver cancer; CNLC, Chinese liver cancer.

**Table 2:** Liver function indexes and tumor markers.

Items	N-CP group (*N* = 74)	MDT-CP group (*N* = 76)	*P* value
ALT (U/L), median (IQR)	31.3 (23.9–44.9)	32.0 (20.6–48.0)	0.987
AST (U/L), median (IQR)	40.5 (29.2–54.0)	40.0 (26.8–53.3)	0.632
ALP (U/L), median (IQR)	101.8 (72.5–157.0)	91.4 (73.5–122.8)	0.228
TBIL (μmol/L), median (IQR)	15.3 (10.5–25.0)	14.8 (10.2–21.2)	0.622
CEA (ng/ml), median (IQR)	4.7 (2.7–6.2)	4.1 (2.9–7.0)	0.545
CA199 (U/ml), median (IQR)	24.9 (14.6–43.7)	24.7 (14.9–41.6)	0.897
AFP (ng/ml), median (IQR)	229.3 (10.4–874.8)	111.1 (9.6–1104.1)	0.912

N-CP, normal care program; MDT-CP, multi-disciplinary team care program; ALT, alanine aminotransferase; IQR, interquartile range; AST, aspartate aminotransferase; ALP, alkaline phosphatase; TBIL, total bilirubin; CEA, carcinoembryonic antigen; CA199, carbohydrate antigen 199; AFP, alpha-fetoprotein.

### Comparison of QoL between groups

**Figure 2:**
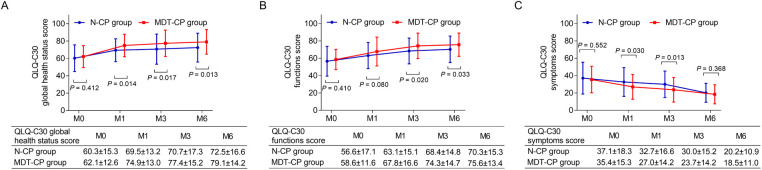
Qol was elevated in MDT-CP group compared to N-CP group. QLQ-C30 global health status score (**A**) and QLQ-C30 functional score (**B**) were elevated, whereas QLQ-C30 symptom score (**C**) was reduced in MDT-CP group compared with N-CP group.

There was no distinction of QLQ-C30 global health status score between MDT-CP group and N-CP group at M0 (*P* = 0.412), while it was higher in MDT-CP group compared to N-CP group at M1 (*P* = 0.014), M3 (*P* = 0.017) and M6 (*P* = 0.013) (Figure 2A). Simultaneously, the QLQ-C30 function score did not differ between groups at M0 (*P* = 0.410) or M1 (*P* = 0.080), whereas it was increased in MDT-CP group compared to N-CP group at M3 (*P* = 0.020) and M6 (*P* = 0.033) (Figure 2B). Regarding QLQ-C30 symptoms score, no gap was found between groups at M0 (*P* = 0.552) or M6 (*P* = 0.368), but it was decreased in MDT-CP group compared to N-CP group at M1 (*P* = 0.030) and M3 (*P* = 0.013) (Figure 2C).

### Comparison of HADS-A score and HADS-D score between groups

**Figure 3:**
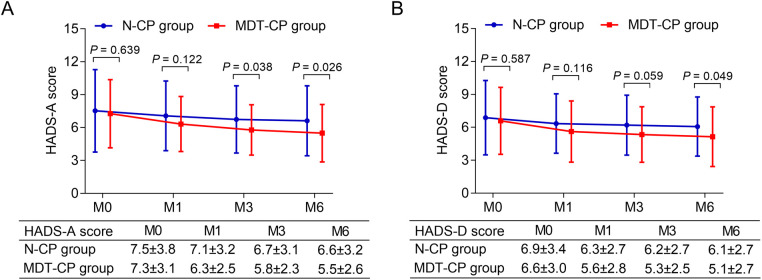
Anxiety and depression were reduced in MDT-CP group compared to N-CP group. HADS-A score (**A**) and HADS-D score (**B**) were decreased in MDT-CP group compared to N-CP group.

HADS-A score was reduced in MDT-CP group compared with N-CP group at M3 (*P* = 0.038) and M6 (*P* = 0.026), but it remained similar between groups at M0 (*P* = 0.639) and M1 (*P* = 0.122) (Figure 3A). In addition, no difference in HADS-D score was found between groups at M0 (*P* = 0.587), M1 (*P* = 0.116) or M3 (*P* = 0.059), however, it was lower in MDT-CP group compared with N-CP group at M6 (*P* = 0.049) (Figure 3B).

### Comparison of anxiety occurrence rate and degree between groups

No difference in anxiety occurrence rate was observed between MDT-CP group and N-CP group at M0 (*P* = 0.643), M1 (*P* = 0.406), or M3 (*P* = 0.196), while it was decreased in MDT-CP group than that in N-CP group at M6 (17.9% vs. 33.3%, *P* = 0.040) (Supplementary Table S1). In addition, there was no distinction of anxiety degree between MDT-CP group and N-CP group at M0 (*P* = 0.580), M1 (*P* = 0.284) or M3 (*P* = 0.117), but it was lower in MDT-CP group than that in N-CP group at M6 (*P* = 0.035) (Supplementary Table S2).

### Comparison of depression occurrence rate and degree between groups

In terms of depression occurrence rate, no difference was found at M0 (*P* = 0.417), M1 (*P* = 0.189), or M6 (*P* = 0.156) between MDT-CP group and N-CP group, whereas it was reduced in MDT-CP group compared with N-CP group at M3 (14.9% vs. 24.6%, *P* = 0.044) (Supplementary Table S3). Besides, there was no difference of depression degree between MDT-CP group and N-CP group at M0, M1, M3, or M6 (all *P* > 0.05) (Supplementary Table S4).

## Discussion

HCC has a profound negative impact on patients’ QoL (27). In addition, the fear of recurrence, especially after surgery or liver transplantation, also seriously affects this (28). Previous studies show that MDT improves QoL of many patients with cancers (18–20, 29), however, the effect of MDT on QoL in HCC patients after surgery is unclear. Our results illustrated that MDT-CP elevated QoL of HCC patients who underwent surgery, especially in terms of global health status and functions, which might be because MDT-CP made a comprehensive and reasonable care plan according to the needs of HCC patients, including (1) Nutrition plans which were formulated by nutritionists to make patients ingest adequate nutrition, and thus improved the patients’ immunity and recovery; (2) Physical training plans according to the patients’ physical conditions, which were made by rehabilitation physicians to enhance the patients’ physical status. Therefore, global health conditions and functions of HCC patients were elevated by MDT-CP. However, comparing with N-CP, the symptoms score in QLQ-C30 was only lower at M1 and M3 by MDT-CP, but there was no difference at M6, which might be because MDT-CP could promote the recovery of patients after surgery in a short time; but with the increase of time, the symptoms of HCC patients in N-CP group were also relieved.

HCC patients who underwent surgery have a high incidence of anxiety and depression (30, 31). One study shows that about 48% of HCC patients who underwent surgery experienced anxiety and about 18% experienced depression (32). This is slightly different from our results, in our study, about 37% of HCC patients developed anxiety and 32% of HCC patients developed depression at M0. The differences of anxiety and depression between the previous study (32) and our study might be due to the differences in assessment populations. The reasons for the high incidence of anxiety and depression in HCC patients after surgery might be that they had enormous pressure, such as worries about their own health status, and a huge financial burden (33, 34). In addition, fear of cancer recurrence was also one of the factors affecting the mental health of postoperative HCC patients. Our study suggested that HCC patients still had a high incidence of anxiety and depression after surgery, therefore, more nursing methods were needed to manage the mental health of HCC patients.

The high incidence of anxiety and depression in HCC patients is a key issue (35). MDT has been shown to alleviate anxiety and depression in patients with various cancers (18–20, 36), while the effect of MDT on mental health in HCC patients is unclear. Our study showed that MDT-CP relieved anxiety and alleviated depression to a certain extent in postoperative HCC patients. This might result from that MDT-CP had a multidisciplinary team of specialists including (1) Professional attending physicians who learned about the condition of disease timely alleviated the patient's fear of recurrence [which was positively associated with the incidence of anxiety and depression (37)]. (2) Psychiatrists who provided psychological interventions on patients relieved their negative emotions. (3) Nursing staffs who provided care to patients during the nursing period might improve their mental health (11). Thus, MDT-CP could relief anxiety and depression of HCC patients.

In the current study, we used a block randomization method with a block size of 4 to randomize 150 patients (21). And there were 38 scratch cards made to record patients’ randomization information. The first 37 scratch cards randomly divided patients into two groups. At this time, there were 74 patients in N-CP and MDT-CP, respectively (148 in total). However, the number of people included was two less than we expected. When the 38th scratch card was opened, two patients just fell into the MDT-CP group. Therefore, there were 76 patients in the MDT-CP group and 74 patients in the N-CP group. This difference in a small number of patients is normal in studies with the block randomization method (38, 39).

There were some limitations in this study: (1) This study was a single-center study, which led to the selection bias. (2) The intervention period was only 6 months; thus, a longer-term intervention was needed to evaluate the effect of long-term MDT-CP on QoL, anxiety and depression in HCC patients. (3) Our study only included HCC patients who underwent surgery. However, many HCC patients were unable to undergo surgery, who even had a poorer QoL and a higher incidence of anxiety and depression. Future studies should elaborate on the effect of MDT-CP in these patients.

In conclusion, MDT-CP improves QoL, mitigates anxiety and depression to a certain degree in HCC patients who underwent surgery. Our findings suggest that MDT-CP could be an effective caring option for the postoperative management of HCC, while a larger-scale study is needed for verification.

## Data Availability

The original contributions presented in the study are included in the article/**Supplementary Material**, further inquiries can be directed to the corresponding author/s.
