# Supplementary material for: Effect of multi-disciplinary team care program on quality of life, anxiety, and depression in hepatocellular carcinoma patients after surgery: A randomized, controlled study
Source: Front Surg. 2023 Jan 6;9:1045003. doi: 10.3389/fsurg.2022.1045003 (PMC9852760; doi:10.3389/fsurg.2022.1045003)
Supplement: Supplementary file 1 [file Table1.docx]

***Supplementary Materials***

**Supplementary Table 1.** Comparison of anxiety occurrence.

| Items | N-CP group | MDT-CP group | *P* value |
| --- | --- | --- | --- |
| Anxiety at M0, No. (%) |  |  | 0.643 |
| No | 45 (60.8) | 49 (64.5) |  |
| Yes | 29 (39.2) | 27 (35.5) |  |
| Anxiety at M1, No. (%) |  |  | 0.406 |
| No | 46 (63.9) | 50 (70.4) |  |
| Yes | 26 (36.1) | 21 (29.6) |  |
| Anxiety at M3, No. (%) |  |  | 0.196 |
| No | 48 (68.6) | 54 (78.3) |  |
| Yes | 22 (31.4) | 15 (21.7) |  |
| Anxiety at M6, No. (%) |  |  | 0.040 |
| No | 46 (66.7) | 55 (82.1) |  |
| Yes | 23 (33.3) | 12 (17.9) |  |

N-CP, normal care program; MDT-CP, multi-disciplinary team care program; M0, month 0 (on discharge); M1, at 1 month after discharge; M3, at 3 months after discharge; M6, at 6 months after discharge.

**Supplementary Table 2.** Comparison of anxiety degree.

| Items | N-CP group | MDT-CP group | *P* value |
| --- | --- | --- | --- |
| Anxiety degree at M0, No. (%) |  |  | 0.580 |
| No | 45 (60.8) | 49 (64.5) |  |
| Mild | 15 (20.3) | 15 (19.7) |  |
| Moderate | 9 (12.2) | 9 (11.8) |  |
| Severe | 5 (6.8) | 3 (3.9) |  |
| Anxiety degree at M1, No. (%) |  |  | 0.284 |
| No | 46 (63.9) | 50 (70.4) |  |
| Mild | 15 (20.8) | 16 (22.5) |  |
| Moderate | 10 (13.9) | 5 (7.0) |  |
| Severe | 1 (1.4) | 0 (0.0) |  |
| Anxiety degree at M3, No. (%) |  |  | 0.117 |
| No | 48 (68.6) | 54 (78.3) |  |
| Mild | 12 (17.1) | 13 (18.8) |  |
| Moderate | 9 (12.9) | 2 (2.9) |  |
| Severe | 1 (1.4) | 0 (0.0) |  |
| Anxiety degree at M6, No. (%) |  |  | 0.035 |
| No | 46 (66.7) | 55 (82.1) |  |
| Mild | 15 (21.7) | 9 (13.4) |  |
| Moderate | 8 (11.6) | 3 (4.5) |  |
| Severe | 1 (1.4) | 0 (0.0) |  |

N-CP, normal care program; MDT-CP, multi-disciplinary team care program; M0, month 0 (on discharge); M1, at 1 month after discharge; M3, at 3 months after discharge; M6, at 6 months after discharge.

**Supplementary Table 3.** Comparison of depression occurrence.

| Items | N-CP group | MDT-CP group | *P* value |
| --- | --- | --- | --- |
| Depression at M0, No. (%) |  |  | 0.417 |
| No | 48 (64.9) | 54 (71.1) |  |
| Yes | 26 (35.1) | 22 (28.9) |  |
| Depression at M1, No. (%) |  |  | 0.189 |
| No | 51 (70.8) | 57 (80.3) |  |
| Yes | 21 (29.2) | 14 (19.7) |  |
| Depression at M3, No. (%) |  |  | 0.044 |
| No | 50 (71.4) | 59 (85.5) |  |
| Yes | 20 (28.6) | 10 (14.5) |  |
| Depression at M6, No. (%) |  |  | 0.156 |
| No | 52 (75.4) | 57 (85.1) |  |
| Yes | 17 (24.6) | 10 (14.9) |  |

N-CP, normal care program; MDT-CP, multi-disciplinary team care program; M0, month 0 (on discharge); M1, at 1 month after discharge; M3, at 3 months after discharge; M6, at 6 months after discharge.

**Supplementary Table 4.** Comparison of depression degree.

| Items | N-CP group | MDT-CP group | *P* value |
| --- | --- | --- | --- |
| Depression degree at M0, No. (%) |  |  | 0.432 |
| No | 48 (64.9) | 54 (71.1) |  |
| Mild | 17 (23.0) | 14 (18.4) |  |
| Moderate | 6 (8.1) | 6 (7.9) |  |
| Severe | 3 (4.1) | 2 (2.6) |  |
| Depression degree at M1, No. (%) |  |  | 0.192 |
| No | 51 (70.8) | 57 (80.3) |  |
| Mild | 16 (22.2) | 11 (15.5) |  |
| Moderate | 5 (6.9) | 2 (2.8) |  |
| Severe | 0 (0.0) | 1 (1.4) |  |
| Depression degree at M3, No. (%) |  |  | 0.060 |
| No | 50 (71.4) | 59 (85.5) |  |
| Mild | 16 (22.9) | 6 (8.7) |  |
| Moderate | 4 (5.7) | 4 (5.8) |  |
| Severe | 0 (0.0) | 0 (0.0) |  |
| Depression degree at M6, No. (%) |  |  | 0.170 |
| No | 52 (75.4) | 57 (85.1) |  |
| Mild | 13 (18.8) | 7 (10.4) |  |
| Moderate | 4 (5.8) | 3 (4.5) |  |
| Severe | 0 (0.0) | 0 (0.0) |  |

N-CP, normal care program; MDT-CP, multi-disciplinary team care program; M0, month 0 (on discharge); M1, at 1 month after discharge; M3, at 3 months after discharge; M6, at 6 months after discharge.
